# Associations between antidepressants and risk of suicidal behavior and violent crimes in personality disorder

**DOI:** 10.1192/j.eurpsy.2025.16

**Published:** 2025-02-03

**Authors:** Kimmo Herttua, Giulio Scola, Tapio Paljarvi, Seena Fazel

**Affiliations:** 1 Department of Public Health, University of Southern Denmark, Esbjerg, Denmark; 2 Department of Psychiatry, University of Oxford, Oxford, UK; 3 Department of Forensic Psychiatry, University of Eastern Finland, Niuvanniemi Hospital, Kuopio, Finland; 4 Oxford Health NHS Foundation Trust, Oxford, UK

**Keywords:** adult psychiatry, antidepressants, forensic psychiatry, personality disorders, suicide & self-harm

## Abstract

**Background:**

Despite uncertain benefits, antidepressants are used in the management of personality disorders (PDs). We investigated the association between antidepressants and two adverse outcomes - suicidal behaviour and violent crimes - in individuals with PDs.

**Methods:**

We used nationwide Danish healthcare registries to identify all individuals with a diagnosed PD aged 18–64 years from 2007 to 2016. Antidepressant use was identified using dispensed prescriptions. Individuals were followed up for healthcare presentations of suicidal behaviour and separately for police-recorded charges of violent crimes. We applied a within-individual design comparing rates of suicidal behaviour and violent crimes during time periods of antidepressant treatment with periods without treatment. Subgroup analyses were performed according to PD clusters, individual antidepressants, specific PDs, psychiatric comorbidities, and history of suicidal behaviour and violent crime.

**Results:**

The cohort included 167,319 individuals with a diagnosed PD, 19,519 (12%) of whom were prescribed antidepressants and presented at least one outcome event during follow-up, making them eligible for within-individual analyses. Overall, we found an association with lower rates of suicidal behavior during periods of antidepressant treatment, compared with periods when individuals were not on antidepressants (incidence rate ratio 0.86, 95% CI 0.84–0.89). However, this association was modified by specific PDs, individual antidepressants, comorbidities, and past history. For violent crimes, we did not observe consistent associations in any direction.

**Conclusions:**

Antidepressants were associated with lower rates of suicidal behaviour, but less clearly in violent crimes. Types of PDs, individual antidepressants, and comorbidities modified these associations.

## Keypoints

**Question** Is treatment with antidepressants associated with suicidal behaviour and violent crimes in individuals with a personality disorder (PD)?

**Findings** In this register-based cohort study of individuals who were diagnosed with a PD in secondary care in Denmark, we used a within-individual design to compare the rates of violent crimes and suicidal behaviour during periods of antidepressant treatment and periods without it. The association between antidepressants and violent crimes was weak. However, we found an overall association between lower rates of suicidal behaviour during treated periods compared to untreated periods. This association between antidepressants and suicidal behaviour was modified by types of PDs and the presence or absence of comorbid psychiatric conditions

**Meaning** Antidepressants may have a role in the management of adverse outcomes in PDs, but this requires consideration of PD types, dimensions, and psychiatric comorbidities

## Introduction

Up to one in every ten adults has a personality disorder (PD) in high-income countries [1]. Individuals with PDs have a higher risk of other psychiatric morbidities, premature mortality, and becoming victims or perpetrators of violence than those without such disorders [2–5].

To date, treatment studies of PDs have primarily focused on reducing mental health symptoms and, to some extent, functional outcomes, such as quality of life [6]. Despite the limited evidence base, antidepressants are used to target various symptoms [7]. For example, in borderline PD (BPD), where most research has been conducted, selective serotonin reuptake inhibitors (SSRIs) are used for emotional dysregulation and poor impulse control [7, 8].

Although antidepressants are used in the management of PDs, their effects on adverse outcomes, specifically suicidal and violent behaviours, remain unclear. Addressing this evidence gap is important for several reasons. Firstly, these medications are increasingly overprescribed in PDs and typically not reviewed regularly, resulting in prolonged exposure [9]. Secondly, PDs are associated with increased risks for these behaviours. According to systematic reviews, individuals with PDs have a threefold increase in the likelihood of violent outcomes compared to the general population [10]. Self-harm and suicide mortality rates are four times higher than those in community controls [11–13]. Finally, these behaviours have serious consequences for public health, including disruptions to healthcare and for caregivers, families, and victims of violence.

Nevertheless, randomized controlled trials in this area are rare. This is partly due to the feasibility challenges of conducting trials involving this patient population [14]. A recent Cochrane review identified only three antidepressant trials with suicide-related outcomes in a total of 103 people with BPD, one of which included suicidal ideation as an outcome [15]. The review reported very low certainty in the evidence.

To address this evidence gap, we used several Danish nationwide registers to examine the association between antidepressant use, suicidal behaviour, and violent crimes among individuals who were diagnosed with PDs. Additionally, we performed subgroup analyses according to the three main PD clusters, individual antidepressants, specific PDs, psychiatric comorbidities, and history of suicidal behaviour or violent crime.

## Methods

### Study design and participants

All data were obtained by linking Danish national registers. The Danish Civil Registration System was established in 1968 and comprises information on all live-born children and new residents in Denmark who are assigned a Civil Personal Register (CPR) number. The CPR number is used to register healthcare services utilization and enables Statistics Denmark to link various data sources at the individual level. All registers have full national coverage, and information from the registers is anonymized when used for research. According to Danish legislation, the use of anonymized national registers for research does not require consent from participants. The authors assert that all procedures contributing to this work comply with the ethical standards of the relevant national and institutional committees on human experimentation and with the Helsinki Declaration of 1975, as revised in 2008. All procedures involving human subjects/patients were approved by the Danish Data Protection Agency and the Danish Health Data Authority (18/16,328).

In this study, participants were restricted to individuals between 18 and 64 years of age during the follow-up period 2007–2016. The follow-up started on January 1, 2007, or on January 1 of the year after participants turned 18. Participants were censored when they reached the age of 65, moved abroad, died, or when the study period ended, whichever happened first. From this full nationwide population sample, we then identified individuals who had been diagnosed with PDs (codes F60.0-F60.9 and F61) using the International Statistical Classification of Diseases and Related Health Problems, Tenth Revision (ICD-10) through the Danish Psychiatric Central Research Register, which contains information on all in- and outpatients for all psychiatric hospitals and outpatient services in Denmark.

### Medications

We extracted data about treatment with antidepressants, identified in the Danish National Prescription Registry according to the Anatomical Therapeutic Chemical (ATC) classification system. These data include all dispensed medication (i.e., prescribed and collected medicines). Antidepressants were defined as drugs with ATC codes N06A. Out of 61,444 individuals who had at least one prescription during the study period, 8,658 (14%) had dispensed tricyclic antidepressants (or non-selective monoamine reuptake inhibitors) (TCAs) (ATC code N06AA), 49,750 (81%) SSRIs (ATC code N06AB), 19,512 (32%) serotonin and noradrenaline reuptake inhibitors (SNRIs), including venlafaxine and duloxetine (ATC code N06AX16 and N06AX21), 22,368 (36%) noradrenergic and specific serotonergic antidepressants (NaSSAs), including mianserine and mirtazapine (ATC code N06AX03 and N06AX11), and 5,121 (8%) other antidepressants. For the analysis, we also extracted information on treatment with antipsychotics (ATC code N05A), hypnotics (ATC code N05C), and anxiolytics (ATC code N05B). Furthermore, for the negative control analysis, we extracted data about treatment with adrenergic inhalants (R03A), a commonly used medication class with negligible psychotropic effects.

### Outcomes

The outcomes of interest were suspicions of violent crimes against an individual (i.e., interpersonal violence, also referred to as violent crimes in this paper) and suicidal behaviour. Data on suspected crimes were extracted from the Administrative System of the National Police, including all crimes and the dates they were reported to the police, even if they were not pursued. A suspected crime follows an initial investigation where a decision is made to pursue a charge. The outcome of suspected crimes, rather than convictions, is more sensitive, as a proportion of such suspicions is dropped based on mental health concerns. Property, traffic, and drug-related offences were excluded.

Data on suicidal behaviour were obtained from the Danish National Patient Register, the Danish Psychiatric Central Research Register, and the Cause of Death Register. Suicidal behaviour refers to suicide attempts and completed suicides. This is in keeping with previous pharmaco-epidemiological studies [16–18]. We identified suicide attempts using a validated algorithm developed using the same registers. The algorithm is based on specific ICD-10 codes for primary or secondary inpatient and outpatient discharge diagnoses from somatic and psychiatric hospitals indicating suicide attempt, intoxication, injury, and self-harm [19, 20]. The following contacts with somatic and psychiatric hospitals were recorded as suicide attempts: (1) all hospital contacts with main ICD-10 diagnosis with codes X60-X84 (intentional self-poisoning and intentional self-harm), (2) all hospital contacts with the reason for contact as NOMESCO (Nordic Medico-Statistical Committee) Code 4 (suicide attempt or self-harm), (3) all hospital contacts with a main diagnosis in chapter F60.0-F60.9 or F61 (PDs) and with concomitant diagnosis of intoxication with codes T36-T50 (all drugs and biological substances, independent of kind of intoxication) and T52-T60 (damaging effect of nonmedical substances, excluding alcohol and food poisoning), and (4) all hospital contacts with a main diagnosis in chapter F60.0-F60.9 or F61 (PDs) and with concomitant diagnosis of cuts to the lower arms, wrists, and hands with codes S51, S55, S59, S61, S65, and S69. Suicide deaths were denoted by suicide recorded as the underlying cause of death on the death certificate.

### Statistical analyses

The primary analyses were conducted using a within-individual design with the 19,519 individuals who had been dispensed antidepressants (prescribed and collected) and presented at least one study outcome. For these analyses, follow-up time was split into treated and untreated periods. An individual was defined as exposed to treatment during the interval between two dispensed antidepressant prescriptions, provided these prescriptions were issued within three months of each other [21]. This is consistent with prescribing practices for these medications. The start of treatment corresponded to the date of the first dispensed prescription, while the end of treatment was defined as the day following three months after the last dispensed prescription. We censored observations at the end of follow-up, time of death, or emigration. Subgroup analyses were stratified by diagnosed psychiatric comorbidities, which included mental and behavioural disorders due to psychoactive substance use (ICD-10 codes F10–F19, i.e., substance use disorders), schizophrenia, schizotypal and delusional disorders (F20–F29, i.e., psychotic disorders), mood disorders (F30–F39), and neurotic, stress-related and somatoform disorders (F40-F48, i.e., anxiety disorders). We also conducted stratified analyses according to the three standard PD clusters: Cluster A (paranoid and schizoid PD); Cluster B (BPD, histrionic, narcissistic, and dissocial PD); and Cluster C (anankastic, anxious, and dependent PD). Furthermore, we stratified individuals based on their history of the selected outcomes, determined by identifying suicidal behaviour or violent crimes in the six years preceding the first dispensed prescription of antidepressants. However, we excluded the last year before the first dispensed prescription to avoid reverse causation bias.

We calculated incidence rate ratios (IRRs) using conditional Poisson regression. In this self-controlled case series (SCCS) analysis, which is a case-only method, the relative risk is based on within-individual comparisons rather than between-individual comparisons, with each individual contributing to both exposed and unexposed observation time [22]. The model, therefore, requires that each individual has at least one exposed and unexposed period. This approach provides implicit adjustment for all observed and unobserved time-invariant characteristics of individuals, such as sex at birth and genetic predisposition, while also allowing control for observed time-varying covariates, such as age [22]. Therefore, the estimated coefficients of these models cannot be biased because of omitted time-invariant characteristics. The within-individual analyses were adjusted for age and co-occurring use of antipsychotics and hypnotics/anxiolytics. However, as antipsychotics and hypnotics/anxiolytics are commonly prescribed for individuals with PDs, we also performed within-individual analyses excluding those with prescriptions for these medications. This SCCS method was also used for the negative control analyses, sensitivity analyses, and analyses for individual antidepressant medication separately, in which we only included individuals who had a dispensed prescription for this single medication in question.

In additional analyses, we fitted a series of longitudinal Poisson regression models to the full cohort of individuals diagnosed with PDs during the ten-year study period (*n* = 167,319). These models were used to analyze between-individual associations, that is, to compare the outcome rates between individuals who had been on antidepressant medication at some point during the study period versus those who had not. These between-individual analyses were adjusted for psychiatric comorbidities, use of antipsychotics and hypnotics/anxiolytics, and age as a continuous variable, which were allowed to vary over time. These analyses were also adjusted for baseline information on educational level and living arrangements. Educational level was based on the highest level of education achieved and was dichotomized into “higher education” (equivalent to bachelor’s level or higher), and “lower education” (equivalent to high school level or lower). Living arrangements were dichotomized into “living with other individuals” and “living alone.” Rates per 1,000 person-years with standard errors were estimated marginal means derived from the full-adjusted Poisson regression models. A *p*-value less than 0.05 was deemed statistically significant. All analyses were conducted using Stata software, version MP 17.0 (Stata Corp., College Station, TX, USA).

### Sensitivity analysis

Attempting suicide or committing a violent crime might increase the probability of subsequent antidepressant treatment. To address this possibility of reverse causality, we excluded crime events or suicide attempts that had occurred between seven and 30 days prior to the start of the antidepressant prescription. We also used the within-individual design for the sensitivity analyses.

## Results

Table 1 shows the baseline characteristics of individuals with diagnosed PDs who were dispensed an antidepressant (*n* = 19,519; AD cohort) and those who were not (*n* = 105,875; non-AD cohort) from 2007 to 2016. We excluded individuals who were prescribed antidepressants but had no outcomes (*n* = 41,925) from this descriptive analysis. Overall, individuals in the AD study cohort were younger, more often women, with lower educational attainments, and more likely to be married or cohabiting than those in the non-AD cohort. Among those with PDs, those prescribed antidepressants were more often diagnosed with Cluster B PD compared to other PD clusters, and they were also more likely to be dispensed with other psychotropic medications. In terms of comorbidities, individuals in the AD cohort were more often diagnosed with substance use, mood disorders, and anxiety disorders, whereas those in the non-AD cohort were more often diagnosed with anxiety, mood, and psychotic disorders. The four most common PD subcategories in both the AD and the non-AD cohorts were BPD (code F60.3), unspecified (code F60.9), anxious (avoidant) (code F60.6), and mixed and other (code F61). Co-occurring categories of PD were more common in the AD cohort than in the non-AD cohort (37% vs. 33%).

**Table 1.** Characteristics of people with a diagnosed personality disorder for those who were included in within-individual analyses (antidepressant or “AD cohort”) and those who had no dispensed prescriptions of antidepressant medicine (“Non-AD cohort”) in 2007–2016 (*n* = 125,394)

	AD cohort	Non-AD cohort	
	*N* (%)	*N* (%)	P value
*Age group, years*			<0.001
18–24	6,720 (34)	25,667 (24)	
25–34	4,819 (25)	22,672 (21)	
35–49	5,632 (29)	33,470 (32)	
50–64	2,348 (12)	24,066 (23)	
*Sex at birth*			<0.001
Men	8,250 (42)	46,576 (44)	
Women	11,269 (58)	59,299 (56)	
*Educational level*			<0.001
Higher	7,890 (40)	49,602 (47)	
Lower	11,629 (60)	56,273 (53)	
*Living arrangement*			<0.001
Married or cohabiting	8,683 (44)	41,114 (39)	
Living alone	10,836 (56)	64,761 (61)	
*Personality disorder*			
Paranoid	632 (3)	3,613 (3)	0.215
Schizoid	645 (3)	4,667 (4)	<0.001
Dissocial	1,391 (7)	7,188 (7)	0.086
Emotionally unstable (borderline)	9,961 (51)	49,078 (46)	<0.001
Histrionic	379 (2)	2,733 (3)	<0.001
Anankastic	537 (3)	2,782 (3)	0.323
Anxious	2,876 (15)	16,237 (15)	0.032
Dependent	1,040 (5)	7,324 (7)	<0.001
Other specific	1,069 (5)	6,986 (7)	<0.001
Unspecified	8,840 (45)	44,456 (42)	<0.001
Mixed and other	3,321 (17)	16,051 (15)	<0.001
Co-occurring (2+) personality DOs	7,309 (37)	35,137 (33)	<0.001
*Personality disorder cluster*			
Cluster A	1,232 (6)	8,028 (8)	<0.001
Cluster B	11,443 (59)	58,477 (55)	<0.001
Cluster C	4,137 (21)	24,360 (23)	<0.001
*Comorbidity*			
Substance use disorders (F10-F19)	5,154 (26)	23,154 (22)	<0.001
Psychotic disorders (F20-F29)	3,944 (20)	25,972 (25)	<0.001
Mood disorders (F30-F39)	8,949 (46)	39,116 (37)	<0.001
Anxiety disorders (F40-F48)	11,780 (60)	51,486 (49)	<0.001
*Medication* *			
Antidepressants	19,519 (100)		
Antipsychotics/mood stabilizers	11,705 (60)	48,300 (46)	<0.001
Hypnotics/anxiolytics	12,979 (66)	53,451 (50)	<0.001
*Total*			
Individuals	19,519 (100)	105,875 (100)	
Person-years at risk	176,638 (100)	880,729 (100)	

The AD cohort refers to individuals with PD who were included in the within-individual analyses (i.e., they had dispensed prescribed antidepressants and had at least one outcome) and the non-AD cohort refers to individuals who had no dispensed prescriptions of antidepressants and were not included in within-individual analyses.

* Number with proportion (%) in medication refers to a number of people that have at least one purchase of the medication in question during the follow-up.

To account for confounders that were time-invariant within each patient during follow-up, we conducted within-individual analyses to compare rates of suicidal behaviour and violent crimes in the same individual while on and off medication (Table 2). When including all individuals we observed lower rates of suicidal behaviour during periods of antidepressant treatment, with an IRR of 0.86 (95% CI, 0.84–0.89) (Model 1, Table 2), but not for the rate of suspected violent crimes (IRR 0.98, 95% CI, 0.88–1.09) (Model 1, Table 2).

**Table 2.** Incident rate ratios (IRR) with 95% confidence intervals (95% CI) derived from within-individual analysis of the association between antidepressants and violent crime suspicions or suicidal behaviour among people diagnosed with personality disorders (*n* = 19,519)

	Violent crime						Suicidal behaviour					
	Model 1			Model 2			Model 1			Model 2		
	Cases	IRR	95% CI	Cases	IRR	95% CI	Cases	IRR	95% CI	Cases	IRR	95% CI
***All*** ***(both with and without comorbidity)***												
No medication (reference)	918	1.00		167	1.00		10,368	1.00		2,273	1.00	
Medication	1,037	0.98	0.88, 1.09	106	0.81	0.60, 1.10	10,337	0.86	0.84, 0.89	1,752	0.84	0.78, 0.90
***With comorbidity***												
*All*												
No medication (reference)	803	1.00		129	1.00		9,374	1.00		1,841	1.00	
Medication	943	1.03	0.92, 1.16	82	0.77	0.55, 1.08	9,351	0.87	0.84, 0.90	1,420	0.88	0.81, 0.96
*History of adverse events*												
No medication (reference)	146	1.00		20	1.00		7,286	1.00		1,408	1.00	
Medication	123	0.85	0.64, 1.14	11	0.45	0.19, 1.07	6,816	0.83	0.80, 0.86	1,034	0.86	0.78, 0.95
*No history of adverse events*												
No medication (reference)	657	1.00		109	1.00		2,088	1.00		433	1.00	
Medication	820	1.07	0.94, 1.21	71	0.85	0.59, 1.22	2,535	0.99	0.92, 1.06	386	0.94	0.79, 1.10
***No comorbidity***												
*All*												
No medication (reference)	115	1.00		38	1.00		994	1.00		432	1.00	
Medication	94	0.58	0.41, 0.83	24	0.99	0.53, 1.85	986	0.85	0.77, 0.95	332	0.68	0.57, 0.81
*History of adverse events*												
No medication (reference)	28	1.00		10	1.00		740	1.00		305	1.00	
Medication	16	0.46	0.17, 1.24	5	0.81	0.19, 3.41	686	0.79	0.64, 0.99	215	0.63	0.51, 0.79
*No history of adverse events*												
No medication (reference)	87	1.00		28	1.00		254	1.00		127	1.00	
Medication	78	0.61	0.42, 0.90	19	1.07	0.53, 2.15	300	0.96	0.67, 1.37	117	0.78	0.57, 1.05

Medication refers to periods on antidepressant medication; no medication to periods without antidepressant medication.

Comorbidity refers to people diagnosed with PDs who have additional diagnosed psychiatric conditions.

History of adverse events refers to people diagnosed with PDs who had a history of suicidal behaviour or violent crimes at least one year before the first antidepressant dispensation.

All models were adjusted for age. Model 1 was additionally adjusted for the use of other psychotropics, including antipsychotics and hypnotics/anxiolytics. In Model 2, people with prescribed antipsychotics and hypnotics/anxiolytics were excluded.

### Subgroup analyses

In models where people with co-prescription of antipsychotics and hypnotics/anxiolytics were excluded (Model 2 in Table 2), we found reduced rates of suicidal behaviour in those with a history of suicidal behaviour, if they had psychiatric comorbidities (IRR, 0.86 [95% CI, 0.78–0.95]) or not (IRR, 0.63 [95% CI, 0.51–0.79]). This association was weaker in those without a history of suicidal behaviour. Conversely, for violent crimes, we did not observe an association when those with prescribed antipsychotics and hypnotics/anxiolytics were excluded.

In subgroup analyses stratified by PD clusters, we observed reduced rates of suicidal behaviour in those with Cluster A or C (IRR, 0.67 [95% CI, 0.56–0.80]) (Table 3). For Cluster B, this association varied by comorbidity. Lower suicidality rates were found for Cluster B without comorbidity (IRR, 0.66 [95% CI, 0.50–0.88]) (Table 3), while no association was found in those with comorbidity.

**Table 3.** Incident rate ratios (IRR) with 95% confidence intervals (95% CI) derived from within-individual analysis of the association between antidepressants and suicidal behaviour stratified by personality disorder cluster

	Suicidal behavior					
	Cluster B			Clusters A and C		
	Cases	IRR	95% CI	Cases	IRR	95% CI
***All** **(both with and without comorbidity)***						
No medication (reference)	973	1.00		422	1.00	
Medication	776	0.91	0.82,1.02	309	0.67	0.56,0.80
***With comorbidity***						
*All*						
No medication (reference)	802	1.00		341	1.00	
Medication	652	0.97	0.86, 1.10	224	0.62	0.51, 0.73
*History of adverse events*						
No medication (reference)	623	1.00		242	1.00	
Medication	495	0.94	0.82, 1.09	164	0.68	0.54, 0.86
*No history of adverse events*						
No medication (reference)	179	1.00		99	1.00	
Medication	157	1.06	0.82, 1.37	60	0.49	0.33, 0.72
***No comorbidity***						
*All*						
No medication (reference)	171	1.00		81	1.00	
Medication	124	0.66	0.50, 0.88	85	0.86	0.59, 1.24
*History of adverse events*						
No medication (reference)	122	1.00		53	1.00	
Medication	84	0.68	0.48, 0.95	44	0.66	0.40, 1.08
*No history of adverse events*						
No medication (reference)	49	1.00		28	1.00	
Medication	40	0.63	0.37, 1.07	41	1.23	0.69, 2.19

Medication refers to periods on antidepressant medication; no medication to periods without antidepressant medication.

Comorbidity refers to people diagnosed with PDs who have additional diagnosed psychiatric conditions.

History of adverse events refers to people diagnosed with PDs who had a history of suicidal behaviour or violent crimes at least one year before the first antidepressant dispensation.

Subgroup analyses were performed based on PD clusters, with Cluster B (emotionally unstable/borderline, histrionic, narcissistic, and dissocial PD) analysed separately, and Clusters A (paranoid and schizoid PD) and C (anankastic, anxious, and dependent PD) combined.

Individuals with prescribed antipsychotics or hypnotics/anxiolytics were excluded from the analyses.

All models were adjusted for age.

**Figure 1.**
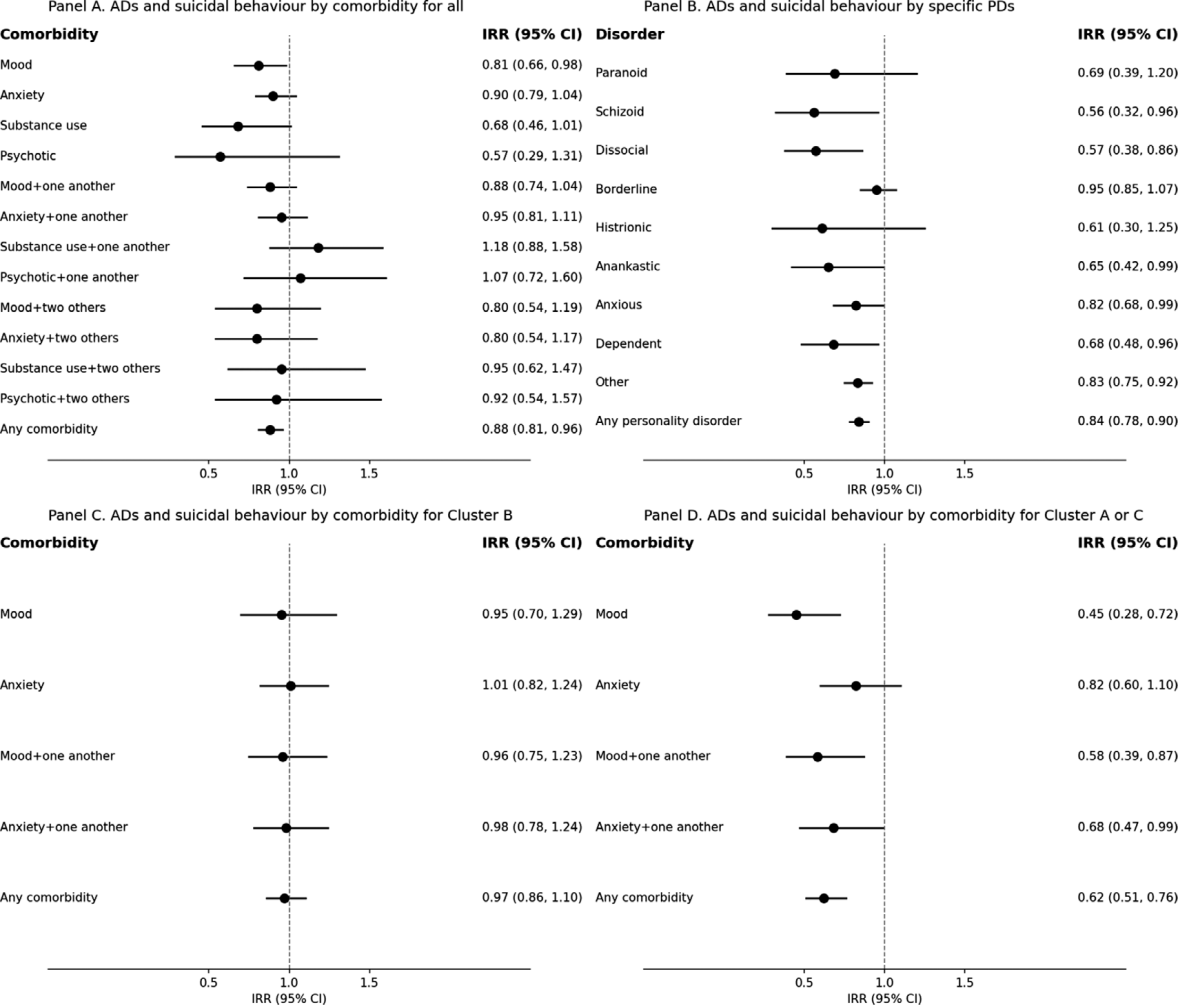
Incident rate ratios (IRR) with 95% confidence intervals (95% CI) derived from age-adjusted within-individual analysis for the association between antidepressants (ADs) and suicidal behaviour stratified by specific PDs, PD clusters, and comorbidity. Individuals with prescribed antipsychotics or hypnotics/anxiolytics were excluded from the analyses. People with a single category (e.g., mood disorder) do not have other diagnosed psychiatric comorbidities.

We further performed subgroup analyses on the association between the use of antidepressants and suicidal behaviour among individuals with diagnosed psychiatric comorbidities, excluding those with prescribed antipsychotics and hypnotics/anxiolytics (Figure 1, Panel A). The overall association with any comorbidity was reduced (IRR, 0.88 [95% CI, 0.81–0.96]). In subgroup analyses for those in Cluster A or C, we observed reduced rates in relation to any comorbidity (IRR, 0.62 [95% CI, 0.51–0.76]), mood disorder (IRR, 0.45 [95% CI, 0.28–0.72]), mood disorder with one another comorbidity (IRR, 0.58 [95% CI, 0.39–0.87]), and anxiety disorder with one another comorbidity (IRR, 0.68 [95% CI, 0.47–0.99]) (Figure 1, Panel D). In contrast, we found no associations by comorbidity for those in Cluster B (Figure 1, Panel C).

### Individual antidepressants

We also performed within-individual analyses for the association between individual antidepressants and suicidal behaviour (Table 4). In Model 1, where specific antidepressants were not mutually exclusive, significant associations were found for all antidepressants except for SNRIs. The associations were strong for TCAs (IRR, 0.68 [95% CI, 0.54–0.85]), other antidepressants (IRR, 0.72 [95% CI, 0.52–0.99]), and NaSSAs (IRR, 0.78 [95% CI, 0.68–0.90]). These results were similar in the analyses where only individuals taking each specific antidepressant type exclusively were included (Model 2 in Table 4), with the upper 95% CI crossing one only for the category “other antidepressants.” In analyses for violent crimes, a significant association was found for NaSSAs (IRR, 0.41 [95% CI, 0.20–0.82]) (data not shown).

**Table 4.** Incident rate ratios (IRR) with 95% confidence intervals (95% CI) derived from within-individual analysis for the association between specific antidepressants and suicidal behaviour among people diagnosed with personality disorders (*n* = 19,519)

	Model 1			Model 2		
	Cases	IRR	95% CI	Cases	IRR	95% CI
*Any antidepressant*						
No medication (reference)	2,273	1.00		1,410	1.00	
Medication	1,752	0.84	0.78,0.90	1,012	0.83	0.75,0.91
*TCAs*						
No medication (reference)	293	1.00		102	1.00	
Medication	157	0.68	0.54, 0.85	34	0.55	0.35, 0.89
*SSRIs*						
No medication (reference)	1,805	1.00		1,024	1.00	
Medication	1,524	0.87	0.80, 0.94	837	0.87	0.78, 0.98
*SNRIs*						
No medication (reference)	494	1.00		86	1.00	
Medication	505	0.94	0.81, 1.09	62	0.80	0.53, 1.19
*NaSSAs*						
No medication (reference)	644	1.00		160	1.00	
Medication	438	0.78	0.68, 0.90	65	0.70	0.49, 0.99
*Other AD*						
No medication (reference)	141	1.00		38	1.00	
Medication	89	0.72	0.52, 0.99	14	0.63	0.29, 1.38

Model 1 included people diagnosed with PDs dispensed more than one type of antidepressant during study period. Model 2 included people diagnosed with PDs dispensed only one specific type of antidepressant during study period. Individuals with prescribed antipsychotics or hypnotics/anxiolytics were excluded from the analyses. Models were adjusted for age.

### Specific PDs

We also conducted within-individual analyses for suicidal behaviour separately among individuals diagnosed with specific PDs, excluding people with prescribed antipsychotics and hypnotics/anxiolytics (Figure 1, Panel B). We observed significant associations for people with schizoid, dissocial, anankastic, anxious, dependent, and other PDs. However, differences in the magnitude of associations across these PDs were relatively small.

### Negative control

We performed within-individual negative control analyses to examine the association between adrenergic inhalants and adverse outcomes (Supplementary Table 1). We did not observe any significant associations between these medications and violent crimes or suicidal behaviour.

### Other statistical approaches

In between-individual analyses, compared with men who had not been dispensed antidepressants, men who had been dispensed antidepressants had an adjusted IRR of 1.16 (95% CI, 1.13–1.18) for suicidal behaviour and 1.07 (95% CI, 1.01–1.13) for violent crimes (Supplementary Table 2). For women, the corresponding IRR was 1.08 (95% CI, 1.06–1.10) for suicidal behaviour and 1.00 (95% CI, 0.90–1.11) for violent crimes. In analyses stratified by comorbidity, men without comorbidities had an IRR of 1.19 (95% CI, 1.02–1.38) for violent crimes, whereas women had an IRR of 1.27 (95% CI, 0.97–1.67). For suicidal behaviour, IRRs were of the same magnitude, regardless of whether men or women had comorbidities.

### Sensitivity analyses

To account for the potential bias induced by reverse causation (e.g., a criminal offence or suicide attempt leading to a new antidepressant prescription), we excluded violent crimes and suicidal behaviour that occurred between seven and 30 days before each medication period. We also excluded individuals with prescribed antipsychotics and hypnotics/anxiolytics. For suicidal behaviour, the patterns of association observed in these models were comparable to those observed in the models without these exclusions (Supplementary Figure 1). The observations for violent crimes were insufficient to perform analyses accurately, but comparable patterns were found when including individuals with prescribed antipsychotics and hypnotics/anxiolytics in the analysis.

## Discussion

In this population-based study of 167,319 people with PDs, we investigated associations between antidepressant treatment, suicidal behaviour, and violent crimes. In the study cohort, 77,015 (46%) had suicidal behaviours, and 11,878 (7%) had suspicions of violent crime over a mean follow-up of 8.5 years. Overall, using within-individual models, we found an overall association between antidepressant treatment and lower rates of suicidal behaviour, but this association was modified by specific PDs, individual antidepressants, the presence of comorbidities, and history of adverse events. For violent crimes, we found no clear overall association in any direction.

In subgroup analyses, the association between antidepressants and suicidal behaviour was stronger in individuals with comorbidity in Cluster A or C PDs, and in Cluster B without comorbidity. In our analysis of the association between individual antidepressants and suicidal behaviour, we observed stronger effects for specific antidepressant classes, including TCAs, NaSSAs, and the category of other antidepressants, though the differences between classes were modest.

To our knowledge, this is the first study to report the association between antidepressants and suicidal behaviour in individuals with PDs. Findings from existing systematic reviews have focused on borderline PD and were based on three small RCTs with inconsistent results [15]. Another systematic review concluded that there have been too few RCTs examining antidepressants in the management of impulsivity [23]. However, another meta-analysis on borderline PD, based on four studies with affective dysregulation as an outcome, found an effect of antidepressants [24]. The latter review suggests that emotional dysregulation could be one mechanism through which antidepressants could reduce suicidal behaviours in some people with PD. In keeping with this, the current study found that individuals with Cluster A or C PDs and with comorbidities, particularly those with mood disorders, had reduced rates of suicidal behaviour during the periods of antidepressant treatment. This may be explained by the specific effects of antidepressants on symptoms linked to suicidality, such as hopelessness, depressed mood, and increased anxiety. Furthermore, in people with Cluster B PDs without comorbidities, antidepressants use was associated with lower rates of suicidal behaviour. Here antidepressants may have effects on symptoms common in Cluster B PDs, especially in BPD, such as affective dysregulation and impulse-behavioural dysfunction.

When examining specific antidepressant classes, TCAs appeared to have stronger associations with suicidal behaviour. TCAs have demonstrated greater efficacy in more severe cases (i.e., hospitalized patients) [25] and an overall stronger efficacy [26], but the risk of overdose mortality and side effects limits their use. This concern is particularly relevant for individuals with PDs, given that prescription medicines are a common means of self-harm and suicide.

For violent crime suspicions, we did not find any clear association with antidepressants. This is partly due to the few high-risk individuals who were only prescribed antidepressants and the likely stronger effects of co-prescribed antipsychotics [16, 27].

In terms of clinical implications, these findings suggest that antidepressant medications may have a role in preventing suicidal behaviour in specific subgroups of people with PDs. But there are two important caveats to this. First, triangulation with other research designs is necessary. To date, trial data, including only a few trials and with very low certainty of evidence, comparing antidepressants with placebo for outcomes of self-harm or suicidal behaviour have shown no clear links in any direction [8, 15]. Second, if antidepressants are prescribed to individuals with PDs, medication reviews are needed every few months so that people do not remain on them unnecessarily for the medium or longer term [9]. As for reducing the risk of violent crimes, our study does not suggest a clear role for antidepressants in individuals with PDs. This is consistent with longitudinal studies of released prisoners, where antidepressants had no associations with reoffending risk using a within-individual design [21]. Rather, other violence prevention strategies should be considered, including the short-term use of antipsychotics [16], treatment of comorbid substance misuse [21], and addressing psychosocial needs.

Our study’s strengths include nationwide high-quality registers, a within-individual design to account for confounding by indication and other unmeasured time-invariant confounders, and the use of information on dispensed prescriptions rather than issued prescriptions. Limitations include those shared with other studies using registers. Firstly, information on dispensed (prescribed and collected) medications does not capture adherence, which is also a limitation in trials and may lead to exposure misclassification bias. However, it is unlikely that this misclassification would be systematic in our data. Secondly, symptom severity in PDs or other undiagnosed mood disorders may introduce reverse causation bias if these first lead to the prescription of antidepressants and then to experiencing the selected outcomes. Since these are time-variant confounders and registers do not include information on the severity of symptoms or undiagnosed conditions, within-individual analyses cannot fully address this. However, since the study’s focus was on antidepressant associations with specific adverse outcomes and not on side effects associated with antidepressants, our results reflect the real-world effectiveness of antidepressants in PDs for these selected outcomes. Thirdly, because administrative registers do not include all potential confounders, our study cannot identify time-varying factors explaining the observed associations. Fourthly, because certain types of PD, such as dissocial PD, may be more reliably diagnosed at older ages, misclassification of PD status could have affected the observed associations in the younger age groups included. Fifthly, while the validated algorithm we used improves the detection of suicide attempts in administrative data, particularly in psychiatric populations such as PDs, where intentionality is often unclear, it may not have captured all suicide attempts and could have led to the inclusion of some non-suicidal self-harm or other injury events as suicide attempts. Finally, the use of clinical diagnoses of PDs rather than standardised diagnostic tools may limit the generalisability of the findings. The most likely consequence is that the included PDs are those that are referred or present to clinical services due to severity and links with suicidal behaviours.

In summary, in this large population-wide study, we demonstrated an overall association between antidepressant use and suicidal behaviours among individuals with PDs. This association was modified by specific PDs, individual antidepressants, presence of psychiatric comorbidities, and history of suicidal behaviours. This suggests individualized treatment that takes into account current specific and concurrent diagnoses, treatment response, and personal preferences, and includes regular review of antidepressant prescriptions.

## Supporting information

Herttua et al. supplementary material 1 Herttua et al. supplementary material

Herttua et al. supplementary material 2 Herttua et al. supplementary material

## Data Availability

Data may be obtained from a third party (the Danish Health Data Authority for health-related data and the National Commissioner of the Danish Police for crime-related data) and are not publicly available.

## Supplementary material

The supplementary material for this article can be found at http://doi.org/10.1192/j.eurpsy.2025.16.
