# Supplementary material for: Associations between antidepressants and risk of suicidal behavior and violent crimes in personality disorder
Source: Eur Psychiatry. 2025 Feb 3;68(1):e28. doi: 10.1192/j.eurpsy.2025.16 (PMC11883785; doi:10.1192/j.eurpsy.2025.16)
Supplement: Herttua et al. supplementary material 1 — Herttua et al. supplementary material [file S0924933825000161sup001.docx]

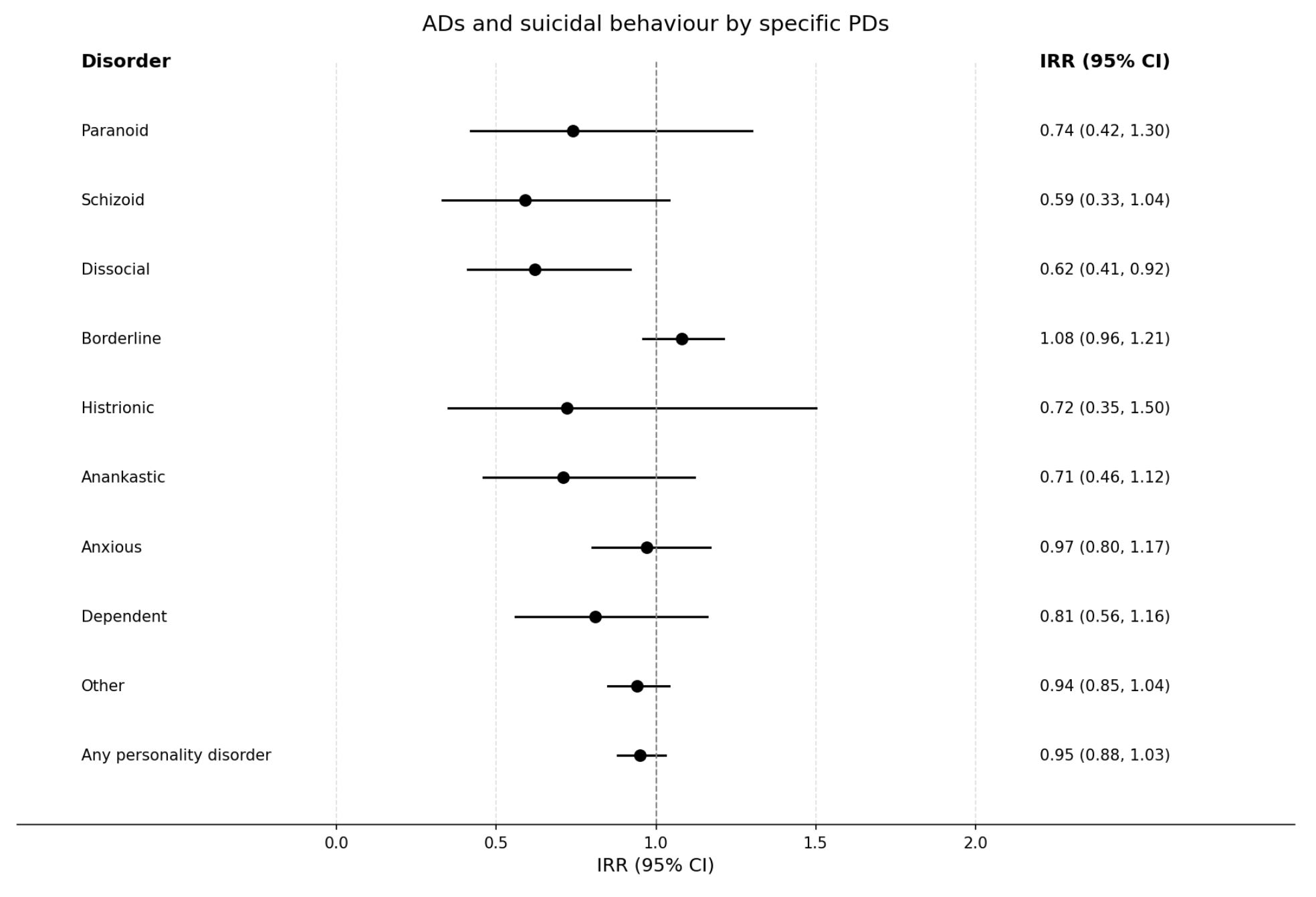


**eFigure 1.** Sensitivity analysis showing incident rate ratios (IRR) with 95% confidence intervals (95% CI) derived from age-adjusted within-individual analysis for the association between antidepressants (ADs) and suicidal behaviour among individuals diagnosed with specific personality disorders. Suicide attempts occurring between 7 to 30 days before the AD exposure were excluded.
