# Supplementary material for: Associations between antidepressants and risk of suicidal behavior and violent crimes in personality disorder
Source: Eur Psychiatry. 2025 Feb 3;68(1):e28. doi: 10.1192/j.eurpsy.2025.16 (PMC11883785; doi:10.1192/j.eurpsy.2025.16)
Supplement: Herttua et al. supplementary material 2 — Herttua et al. supplementary material [file S0924933825000161sup002.docx]

| **eTable 1**. Negative control analysis. Incident rate ratios (IRR) with 95% confidence intervals (95% CI) derived from within-individual analysis for the association between adrenergic inhalators and violent crime suspicions or suicidal behaviour among patients diagnosed with personality disorders (n=2,070). | | | | | | | |
| --- | --- | --- | --- | --- | --- | --- | --- |
|  |  | Violent crime | | | Suicidal behaviour | | |
|  |  | Cases | IRR | 95% CI | Cases | IRR | 95% CI |
| Men | |  |  |  |  |  |  |
| No medication (reference) | | 64 | 1.00 |  | 342 | 1.00 |  |
| Medication | | 25 | 0.74 | 0.42, 1.28 | 275 | 1.04 | 0.85, 1.27 |
| Women | |  |  |  |  |  |  |
| No medication (reference) | | 57 | 1.00 |  | 582 | 1.00 |  |
| Medication | | 24 | 1.04 | 0.61, 1.80 | 430 | 1.09 | 0.93, 1.28 |
|  |  |  |  |  |  |  |  |

Models were adjusted for age.

| **eTable 2.** Association between antidepressant medication and violent crime suspicions and suicidal behaviour in personality disorders as reported by incident rate ratios (IRR) with 95% confidence intervals (95% CI) derived from between-individual analyses (n=167,319). | | | | | | | | |
| --- | --- | --- | --- | --- | --- | --- | --- | --- |
|  | Violent crime | | | | Suicidal behaviour | | | |
|  | Cases | Rate (SE) | IRR | 95% CI | Cases | Rate (SE) | IRR | 95% CI |
| *All* |  |  |  |  |  |  |  |  |
| Men |  |  |  |  |  |  |  |  |
| No medication | 14,815 | 39.7 (0.7) | 1.00 |  | 44,100 | 121.6 (1.0) | 1.00 |  |
| Medication | 10,074 | 42.4 (1.0) | 1.07 | 1.01, 1.13 | 31,759 | 140.4 (1.4) | 1.16 | 1.13, 1.18 |
| Women |  |  |  |  |  |  |  |  |
| No medication | 2,595 | 5.3 (0.2) | 1.00 |  | 56,837 | 118.0 (0.8) | 1.00 |  |
| Medication | 1,957 | 5.3 (0.2) | 1.00 | 0.90, 1.11 | 42,759 | 127.3 (1.0) | 1.08 | 1.06, 1.10 |
| *With comorbidity* |  |  |  |  |  |  |  |  |
| Men |  |  |  |  |  |  |  |  |
| No medication | 12,364 | 40.5 (0.8) | 1.00 |  | 39,688 | 131.7 (1.1) | 1.00 |  |
| Medication | 8,788 | 42.4 (1.0) | 1.05 | 0.99, 1.11 | 29,467 | 153.1 (1.6) | 1.16 | 1.13, 1.19 |
| Women |  |  |  |  |  |  |  |  |
| No medication | 2,366 | 5.9 (0.2) | 1.00 |  | 51,242 | 128.5 (1.0) | 1.00 |  |
| Medication | 1,716 | 5.7 (0.2) | 0.97 | 0.87, 1.08 | 38,345 | 137.8 (1.2) | 1.07 | 1.05, 1.10 |
| *No comorbidity* |  |  |  |  |  |  |  |  |
| Men |  |  |  |  |  |  |  |  |
| No medication | 2,451 | 35.4 (1.6) | 1.00 |  | 4,412 | 66.6 (1.3) | 1.00 |  |
| Medication | 1,286 | 42.0 (2.8) | 1.19 | 1.02, 1.38 | 2,292 | 76.7 (2.2) | 1.15 | 1.07, 1.23 |
| Women |  |  |  |  |  |  |  |  |
| No medication | 229 | 2.8 (0.3) | 1.00 |  | 5,595 | 68.2 (1.2) | 1.00 |  |
| Medication | 241 | 3.6 (0.4) | 1.27 | 0.97, 1.67 | 4,414 | 76.9 (1.6) | 1.13 | 1.07, 1.19 |
|  | | | | | | | | |
| Medication refers to individuals that had at least one dispensed antidepressant during the follow-up, while those in the no medication group did not have any dispensed antidepressants. Models were adjusted for age, education, living arrangement, and use of antipsychotics and hypnotics/anxiolytics. Models for All were additionally adjusted for other psychiatric comorbidities. | | | | | | | | |
